# A synbiotic approach to the formulation of innovative bioherbicides for weed control: a global perspective

**DOI:** 10.3389/fpls.2026.1724814

**Published:** 2026-04-13

**Authors:** Hary L. Razafindralambo, Nicolas Mathot, Marie-Laure Fauconnier, M. Haïssam Jijakli

**Affiliations:** 1 BioEcoAgro Joint Research Unit, Teaching and Research Centre (TERRA) Teaching and Research Centre, Laboratory of Microbial Processes and Interactions, Gembloux Agro-Bio Tech, Liege University, Gembloux, Belgium; 2 Integrated and Urban Plant Pathology Laboratory, Gembloux Agro-Bio Tech, Liege University, Gembloux, Belgium; 3 ProBioLab, Namur, Belgium; 4 Laboratory of Chemistry of Natural Molecules, Gembloux Agro-Bio Tech, Liege University, Gembloux, Belgium

**Keywords:** agroecological technique, botanicals, microbes, nature-based herbicides, secondary metabolites, synergism

## Abstract

Effective weed management remains a crucial concern in agriculture. The quest for alternatives to conventional herbicides, driven by limitations and drawbacks, presents a challenge today in terms of efficacy, costs, safety, and weed resistance. Accordingly, new strategies of integrating multiple approaches are emerging. Within this landscape, the utilization of bioherbicides, sourced from microorganisms or plants, holds a prominent place. This perspective paper proposes and discusses the potential of an innovative approach that combines live microorganisms and botanical components in synbiotic formulations to develop the next generation of bioherbicides as a promising solution for sustainable weed management. Their mixture may provide superior efficacy than when each is used individually, due to synergistic interactions arising from complementary and/or cooperative effects. It also addresses strategy design, formulation, and product control while presenting the challenges and potential risks of such concept.

## 1 Introduction

**Figure 1:**
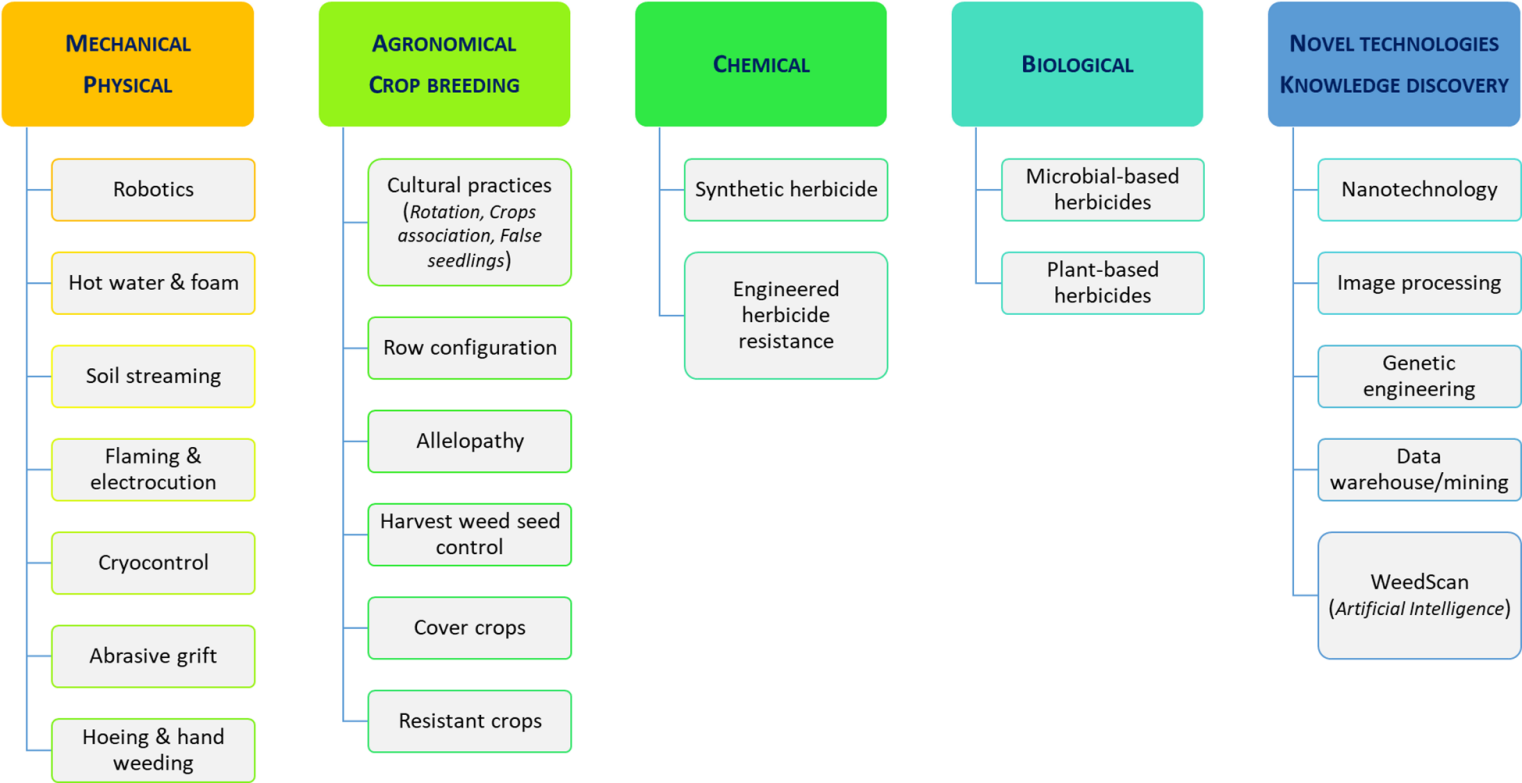
Principal weed management techniques and methods.

Weeds are undesirable competitor plants that severely reduce crop yield in agricultural fields, thereby threatening global food production and natural ecosystems (Duke et al., 2022**;** Kubiak et al., 2022**;** Jiang et al., 2023). It has been estimated that weed species cause the most important crop losses in world agriculture compared to other pests (Bailey, 2014). The selection of effective control strategies is therefore essential and remains a major challenge today, owing to the limitations and drawbacks associated with existing methods such as cultural, physical, mechanical, chemical, and biological techniques (**Figure 1**), either individually or in an integrated management way (Lamberth et al., 2013**;** Radhakrishnan et al., 2018**;** Korres et al., 2019**).** Among the most common practices for weed management control is the use of chemical herbicides such as atrazine, glyphosate, and paraquat, which are efficient compounds in controlling a wide range of weed species germination and growth, but also increase the cases of herbicide resistance agricultural weeds (e.g., *Amaranthus palmeri*, *Conyza bonariensis*, *Lolium rigidum*, and *Avena fatua*) after a long term of applications (Ofosu et al., 2023) and the loss of efficiency (Akhter et al., 2023). Nowadays, there are 273 resistant weed species globally with 57% dicots vs 43% monocots (Heap, 2026). In addition, these synthetic herbicides are sources of water and land contamination, which have negative effects on the environment, as well as on animal and human health. Consequently, there is an urgent need to ensure long-term and sustainable solutions for agricultural and food security in both developed and developing countries (Korres et al., 2019).

The use of nature-based herbicides, known as bioherbicides, is among the emerging methods of weed control that aligns with the sustainable development goals established by the United Nations, contributing, for instance, to responsible consumption and production (SD12) and good health and well-being (SD3) (Uludag et al., 2018**;** Campos et al., 2023).

Bioherbicides consist of living organisms, their derivatives, plant extracts, and allelochemicals that exhibit biological activity in controlling weeds (Zhang et al., 2025). They can mainly be categorized in: (a) microbial-based herbicides, comprising live microorganisms and/or their metabolites, and (b) plant-based herbicides such as pure compounds isolated from plants, plant extracts, and essential oils (Campos et al., 2023). Their mechanisms of action are related to several processes and depend on both the active ingredients and the weed species (Bordin et al., 2021). Microbial bioherbicides (commercialized or not) belong to phytopathogenic and non-phytopathogenic bacteria such as *Xanthomonas* sp. (Camperico^®)^ and *Pseudomonas* sp. (D7^®^), *Streptomyces* sp.), phytopathogenic fungi (e.g., *Colletotrichum* sp./ (Collego)^®^, *Alternaria* sp., *Phoma* sp. (Phoma™), non-phytopathogenic fungi e.g., (*Trichoderma* sp.), obligate fungal parasites e.g., *Puccinia* spp. (Dr. BioSedge^®^), and viruses (SolviNix™) (Kremer, 2005**;** Duke et al., 2022). On the other hand, bioherbicides from plant sources are molecules with well-defined chemical structures, belonging among others to phenolic compounds (e.g., flavonoids, coumarins, quinones), terpenoids (e.g., mono-, sesqui-, di-, & triterpenoids), nitrogen-containing compounds (alkaloids, nonprotein amino acid, benzoxazinoids & cyanogenic glycosides), or constituted by a mixture of compounds such as the case of essential oils that can be obtained by various extraction techniques (Kostina-Bednarz et al., 2023).

Bioherbicides are expected to offer several advantages such as safety, eco-friendliness, higher biodegradability, and multiple modes of action, which could reduce the risk of developing herbicide-resistant weeds compared to synthetic herbicides (Muñoz et al., 2020). New formulations of microbial- or plant-based bioherbicides have often been studied and developed individually rather than in combination for managing weeds. However, a bioherbicide system combining microorganisms and plant extracts can offer additional benefits over individual components in terms of diversity and biological activity, particularly when the active compounds from microbial and plant sources act in synergism. Such hybrid microbe-plant systems, using two component categories from natural sources in one entity, can generate a wide range of herbicidal activities of next generation to control a large variety of weeds.

This 2-in-1 concept combining living microorganisms and non-living substances of plant sources in synbiotics has been successfully applied for human and animal health applications, but less developed for crop protection (Swanson et al., 2020). During the last decades, synbiotics have known a growing interest as functional ingredients in food and feed sectors for promoting health (Song et al., 2012). They have also become a focus of interest for plant preservation/biocontrol and growth, owing to their potential biopesticide and biofertilizer activities, respectively (Shaw and Arnold, 2002**;** Kouhounde et al., 2022). However, this approach has rarely been applied in the development of bioherbicides. Even though synthetic herbicides like glyphosate have been mixed with microbial herbicides to produce synergistic effect, the combination with natural-derived compounds has received little consideration (Gonzini et al., 1999**;** Peng and Wolf, 2011). Among the most challenging steps of bioherbicide development are microbial safety, the concentration and/or purification of raw material extracts, as well as the choice of enhancers and stabilizing adjuvants used in formulation. This aims at ensuring sufficient herbicidal activity, long-term stability, and maintaining such functionalities under a wide range of environmental conditions of water stress and temperature, while guaranteeing human and environmental safety. Another challenge remains the optimization of scale-up processes towards viable industrial scale implementation.

This perspective article introduces and discusses the potential of developing synbiotic-based bioherbicides as a promising solution for sustainable weed control. It also addresses strategy design, formulation, and product assessment while presenting the challenges and potential risks of such approach.

## 2 The emergence of synbiotics

### 2.1 Definition and concept

Initially, a synbiotic was defined as a mixture of probiotic and prebiotic, combining live microorganism and fermentable non-digestible food ingredients that beneficially affect the host (Kolida and Gibson, 2011). More recently, a larger definition has been provided by the International Scientific Association for Probiotics and Prebiotics (ISAPP), as follows: “ A synbiotic is a mixture comprising live microorganisms and substrate(s) selectively utilized by host’s microorganisms that confers a health benefit on the host” (Swanson et al., 2020). In this context, ‘Host microorganisms’ include both autochthonous microorganisms (i.e., resident or colonizing the host) and allochthonous microorganisms (i.e., externally applied such as probiotics). The microbial and substrate components of a synbiotic may not necessarily benefits on the target host, but their mixture should be. Two types of synbiotic have been defined according to the interactions among components (Kolida and Gibson, 2011). When the two components act independently without surpassing effectiveness compared to the simple sum of each, the bioactive synbiotic is known as complementary. Conversely, when there are mutual interaction and/or cooperation between the two components to produce superior effects than those of each component, it is considered as a synergistic synbiotic.

### 2.2 Synbiotics as bioherbicides

The idea to combine beneficial microbes with plant-based bioherbicide in synbiotic is new, or at least rarely exploited in the agronomic sector, according to the available scientific papers and patents. One of the interesting examples reported in literature was the effect of plant-derived components such as mannoses and oxalic acid in enhancing microbial virulence of *Colletotrichum coccodes* through their functionality as plant defense inhibitors (Ahn et al., 2005). Another relevant example was the total herbicide effect of a fungus (*Alternaria crassa*)/pectin mixture on different weeds tested in greenhouse (Boyette and Abbas, 1994). Also, *Trichoderma koningiopsis* was efficiently associated with commercial formulations of glyphosate in controlling weeds and soybean plants (Ulrich et al., 2023).The only patented example was a formulation combining an essential oil component (e.g., eugenol) and a mixture of microorganisms (e.g., *Streptomyces* and *Bacillus* genera) for targeted delivery and controlled release to enhance control of various plant species in a stable and scalable manner (Frank et al., 2023).

Although numerous microorganisms with biological weeding activity have been studied and developed (**Table 1**), only a limited number have been commercialized. This is mainly due to a narrow spectrum in weed control and a sensitivity to climate conditions, as well as a difficulty in scaling-up production. Most of them belong to fungi, particularly the genera *Colletotrichum*, *Fusarium*, *Altenaria*, *Cercospora*, and *Puccinia*. Bacterial bioherbicides include, for instance, the genera *Pseudomonas*, *Enterobacter*, *Flavobacterium*, *Xanthomonas*, and some *Lactobacillus* strains. Their weed control mechanisms rely on phytotoxic metabolites with varied chemical structures, including organic acids, peptides, phenol compounds, and others (e.g., Thaxtomin). These metabolites directly affect specific plant components, disrupting biosynthetic pathways, membrane receptors, proteins, enzymes, and energy metabolism in different ways (Cordeau et al., 2016**;** Fang et al., 2022**;** Cai et al., 2023).

**Table 1:** A list of potential and commercialized microbial bioherbicides and their target weeds.

Bioherbicide agent	Active ingredients/mode of action	Target weed	Commercial status	Reference
Bacterial agents				
*Enterobacter* sp.	*Indole-3- acetic acid (high concentration)*	*Echinochloa crus-galli*; *Portulaca oleracea* (seed germination)	*Not yet*	(Radhakrishnan et al., 2018)
*Xanthomonas campestris*	*Phytopathogenic interactions*	*Poa annua Annual bluegrass*	*Camperico^®^*	(Bailey, 2014)
*Lactic acid bacteria*	*Lactic acid* *Citric acid*	*Trifolium repens; Trifolium pretense; Lotus corniculatus; Medicago lupulina; and Oxalis acetosella*	*KONA*	(Cordeau et al., 2016)
*Streptomyces acidiscabies*	*Thaxtomin A*	*Digitaria sanguinalis; Sorghum bicolor; Solanum nigrum*	*Not yet*	(King et al., 2001)
*Bacillus wiedmannii*	*Cry family proteins (Cry10 Aa, Cry4 Ba, and Cry4 Aa)*	*Lolium temulentum L.*	*Not yet*	(Eigharlou et al., 2024)
*Pseudomonas fluorescens*	*Phytotoxin complex (peptides, organic acids, and lipopolysaccharides)*	*Bromus tectorum (Downy brome)*	*D7^®^*	(Kennedy, 2018)
Fungal agents				
*Trichoderma koningiopsis*	*Spores and hydrolytic enzymes, koninginins*	*Euphorbia heterophylla; Brachiaria plantaginea*	*Not yet*	(Ulrich et al., 2023)
*Albifimbria verrucaria*	*Spores, mycelial fragments, verrucarin A, curvicolides*	*Glyphosate-Resistant Conyza canadensis (horseweed)*	*Not yet*	(Hoagland et al., 2023)
*Bipolaris yamadae*	*Spores (conidies), mycelium*	*Echinochloa crus-galli - Gramineous weeds - Grass weeds in arable crops*	*Not yet*	(Tan et al., 2024)
*Chondrostereum purpureum*	Mycelium	*Stranglervine*	Chontrol™	(De Jong, 2000)
*Sclerotinia minor*	Mycelial growth	*Dandelion & other broadleaf weeds*	SARITOR	(Shaheen et al., 2010)

Concerning plant-based bioherbicides, also known under the terminology botanical herbicides (botanicals), they are defined as single compounds, bioactive mixtures or extracts from plant materials (Duke et al., 2022**;** Fang et al., 2022). Beyond their natural occurring and rapid biodegradation, botanical herbicides have multiple action modes while exerting low toxicity to nontarget species. A large category of botanical herbicides has been widely described in the review paper on the status and prospects botanical biopesticides (Acheuk et al., 2022). Among the most important plant sources of botanical herbicides obtained by extraction are (1) *Eugenia caryophyllus* or *Syzygium aromaticum* for β-caryophyllene, eugenol, and eugenol acetate, (2) Cymbopogon for citronellal, geraniol and citronellol, (3) Cinnamomum for eugenol and trans-cinnamic aldehyde, (4) Eucalyptus for β-triketones, especially leptospermone, (5) Ocimum with geranial and geraniol, linalool, methyl cinnamate and methyl eugenol, (6) Pelargonium for pelargonic acid, (7) Thymus for carvacrol and thymol, (8) Origanum for γ-terpinene and thymol, and (9) Lavandula with fenchone (Campos et al., 2023). **Table 2** illustrates the structural diversity of secondary metabolites with herbicidal activities from microbial and plant origins.

**Table 2:** Examples of microbial and plant phytotoxins.

Chemical class	Chemical structure	
Microbial source (bacteria and fungi)		
Peptides/amino acid-based compounds (Cai et al., 2023)	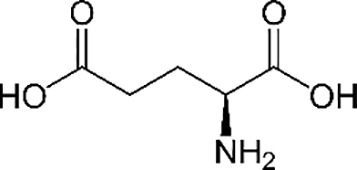	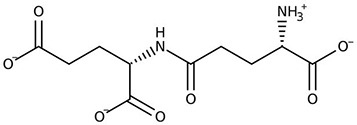
	Glutamic acid	L-glutaminyl-glutamine
Terpenoids (Yan et al., 2018; Yin et al., 2020)	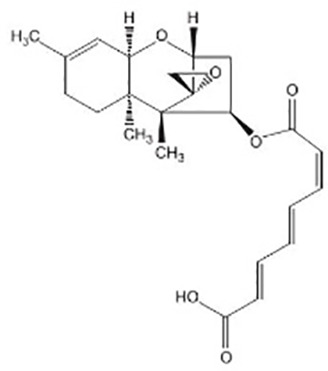	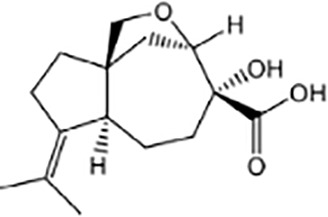
	Harzianum B	Aspterric acid
Macrocidins (Graupner et al., 2003)	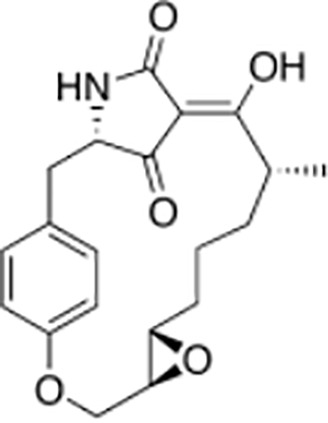	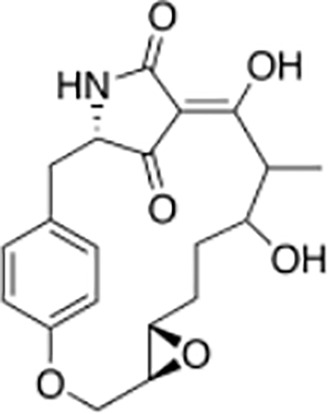
	Macrocidin A	Macrocidin B
Phenolic compounds (Gealy et al., 1996; Adetunji et al., 2019)	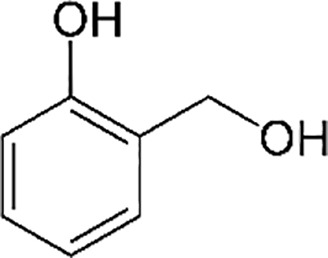	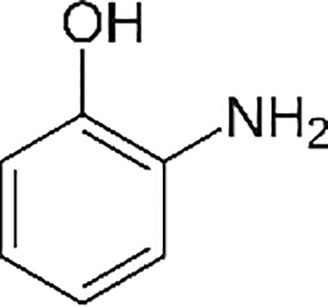
	2-hydroxymethyl phenol	2-amino phenol
Botanical source (single and mixture)		
Alkaloids (Zasada et al., 2012; Dayan et al., 2015)	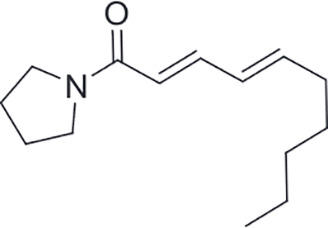	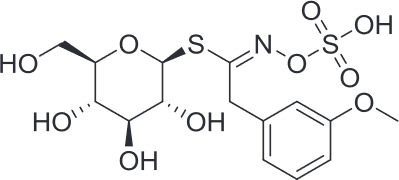
	Sarmentine	Glucolimnanthin
Terpenoids (Verdeguer et al., 2020)	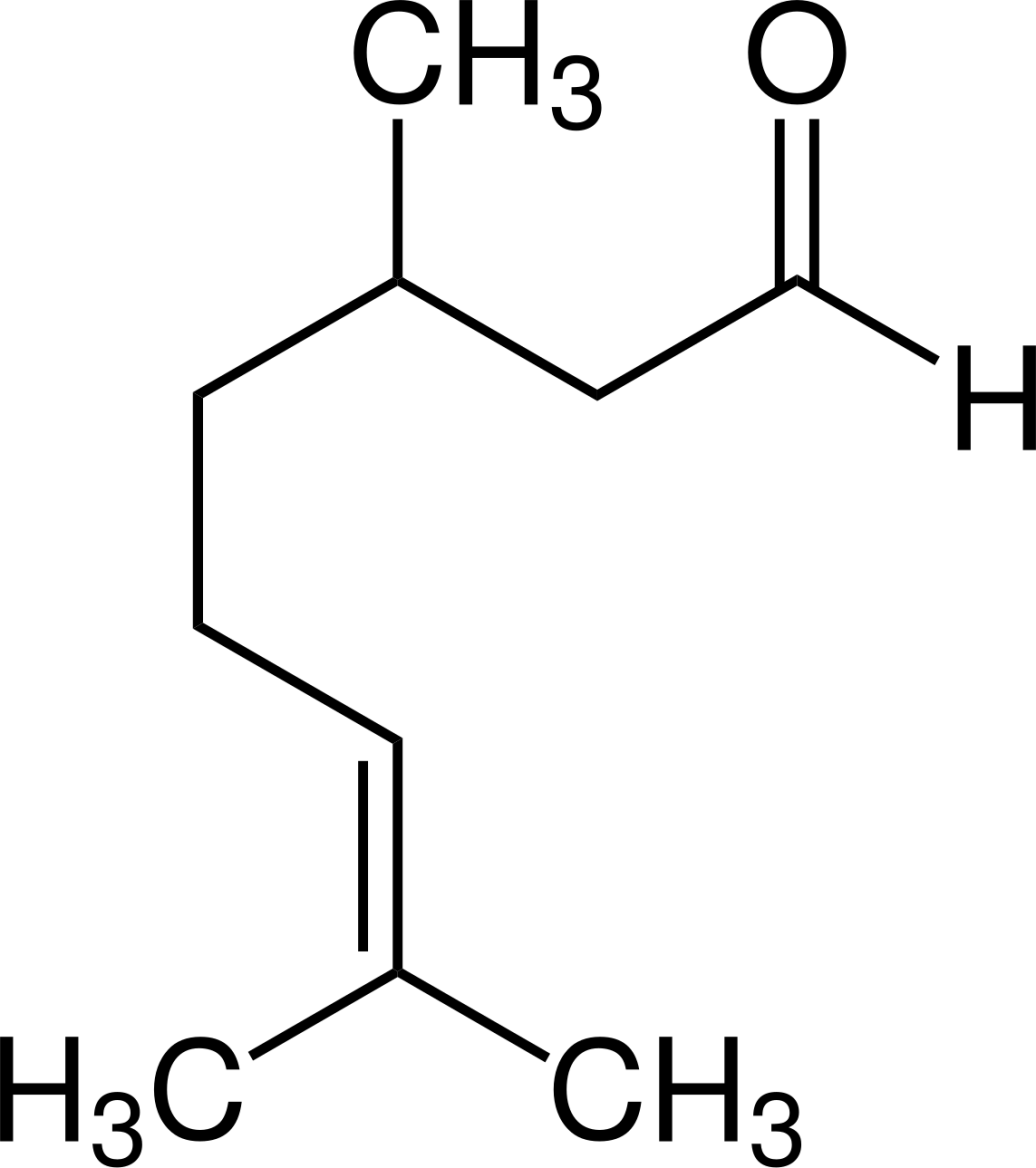	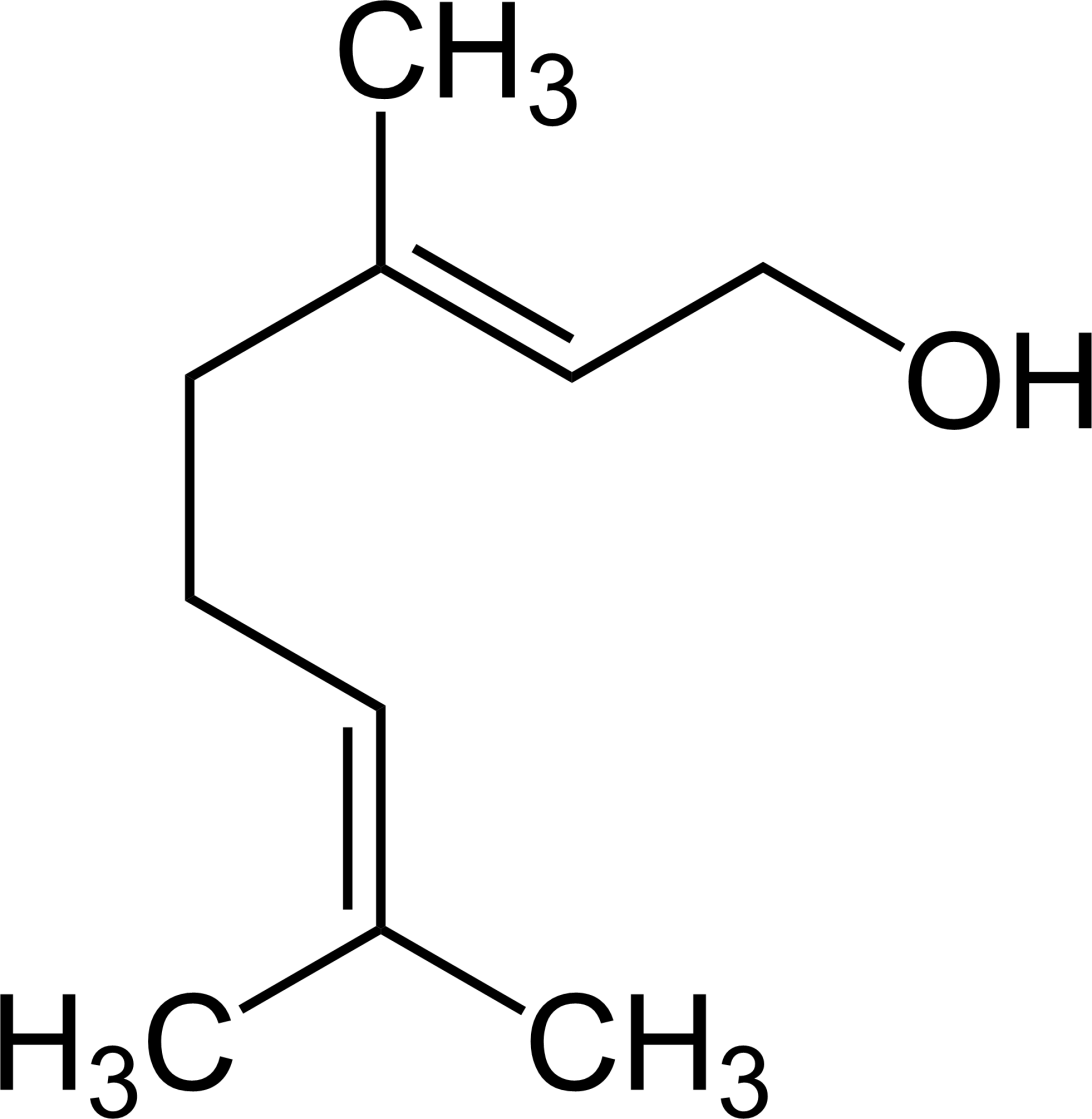
	Citronellal	Geraniol
Polyphenols (Ben Kaab et al., 2020)	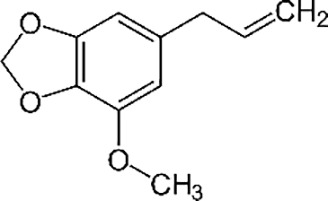	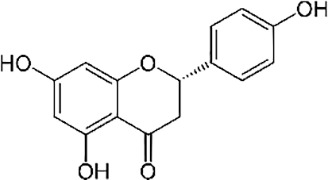
	Myritricin	Naringenin
Organic/Fatty acids (Cai et al., 2023)	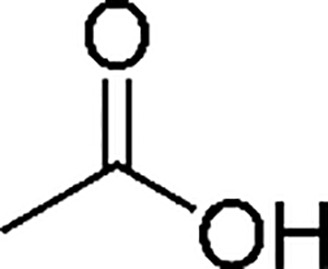	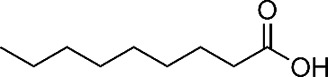
	Acetic acid	Pelargonic acid
Essential oil constituents (Tworkoski, 2002; Fu et al., 2019)	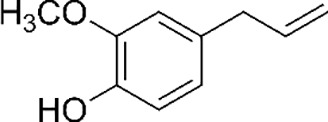	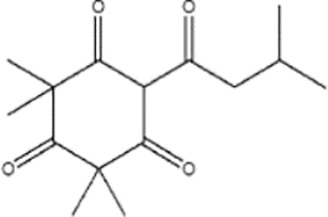
	Eugenol (Clove)	Leptospermone (Manuka)

### 2.3 Mechanisms of action

Microbial and botanical-based bioherbicides exert herbicidal activities against weeds through diverse mechanisms, including plant-pathogen interactions, allelochemicals with direct phytotoxic properties, metabolic disruptions (e.g., enzyme inhibition), hormonal imbalances, cell wall and membrane damage, photosynthesis inhibition, oxidative stress, and mitosis disruption, which are overviewed in the next sections.

#### 2.3.1 Microbial bioherbicides

Live microbial agents can infect target plants through direct or indirect virulence processes. They produce enzymes such as pectinases, cellulases, and ligninases to degrade plant cell walls, as well as proteases, peptidases, amylases, and phospholipases to break down proteins and lipid membranes. These enzymatic activities help facilitate the colonization of plant tissues (Harding and Raizada, 2015).

Additionally, microbes can produce phytotoxic secondary metabolites (toxins) that interfere with plant metabolism by disrupting essential physiological processes and ultimately leading to plant damage or death (Ghorbani et al., 2005).

Other microbial biochemical compounds (e.g., phosphinothricin/glufosinate, bialophos, leptospermone = triketone) target many molecular sites in weeds. Their efficiency is dependent on the bioactive compound specificity and virulency, but also on the co-formulants, dose, phenological stage of the target, and environmental factors. In particular, the plant-associated bacteria (PAB) have different modes of action (Fang et al., 2022):

1. Production of phytotoxic metabolites such as thaxtomins (*Streptomyces* sp.), tagetitoxins (*Pseudomonas syringae*), quinoline (*Pseudomonas aeruginosa* H6) and its derivatives, diketopiperazines (*Bacillus velezensis* JTB8-2), lipopeptides (*Bacillus clausii*), hydrogen cyanide (*Rhizobacteria* sp.).
2. Production of exopolysaccharides (Rhizobacteria isolated from isolated form *Euphorbia* spp.) reducing the growth of leafy spurge calluses.
3. Overproduction of auxins (e.g., 72 mg/L) that inhibit instead of promoting plant growth.
4. Disruption of biochemical pathways that may interfere with crucial enzymatic activities, induce hormonal imbalance, or inhibit metabolic processes (e.g., gibberellin pathway inhibition), provoking weed suppression.
5. Other unclear modes such as provoking oxidative stress, producing volatile ammonia, altering the soil microbial diversity nearby, combination effects of glycine and cyanogenic rhizobacteria, and so on.

In the case of fungi-based bioherbicides, various phytotoxins such as tentoxin (a cyclic tetrapeptide), cornexistin, and macrocidin A are the active agents exhibiting detrimental effects on weeds (Golijan et al., 2023). Among these are the inhibition of seed germination and plant growth, disturbance of photosynthesis, overproduction of reactive oxygen species, or the appearance of necrosis zones. An interesting aspect of some fungi is the production of pectinase that may exhibit the ability to breach the cellular barriers of weed plants through the enzymatic degradation of polysaccharide layers (Hoagland et al., 2023). This mode of action could potentially facilitate the entry of other agents, making it particularly intriguing for synbiotic associations.

In the case of fungi-based bioherbicides, various fungi produce metabolites and enzymes capable of impairing weed establishment and growth through diverse biological mechanisms (Hoagland et al., 2023). Fungal phytotoxins are generally classified as host-selective toxins (HSTs) and non-host-selective toxins (NHSTs). HSTs are active only against susceptible host plants and are typically considered key pathogenicity factors, as their production is often essential for fungal virulence and disease development. In these systems, a close relationship exists between toxin production and pathogenicity in the fungus, and between toxin sensitivity and disease susceptibility in the plant, providing strong evidence that HSTs govern host-selective infection (Tsuge et al., 2013**).** By contrast, NHSTs are not primary determinants of host range and are not indispensable for pathogenicity, although they may substantially contribute to fungal virulence. Owing to their broader spectrum of activity, NHSTs are of particular interest for weed management, as they can affect not only the host of the producing fungus but also a range of non-host plant species. Their phytotoxic effects include inhibition of seed germination and plant growth, disruption of photosynthesis, induction of reactive oxygen species, and necrosis formation. Examples include cornexistin, which has been investigated as herbicidal compound, and tentoxin, a cyclic tetrapeptide produced by *Alternaria alternata* that interferes with chloroplast development (Pusztahelyi et al., 2015). In addition to toxin production, some fungi secrete cell wall-degrading enzymes, notably pectinases, which may facilitate penetration into weed tissues by degrading polysaccharide barriers (Hoagland et al., 2023). This enzymatic activity may also enhance the entry or efficacy of other bioactive compounds, making fungi particularly attractive candidates for integrated or synbiotic weed management strategies.

#### 2.3.2 Botanical bioherbicides

Botanical-based bioherbicides are bioactive mixtures, extracts, and active compounds or allelochemicals from plant materials (e.g., leaves, roots, or seeds). Their mechanisms also involve plant-pathogen interactions or allelopathic compounds through interference with various physiological processes in weeds. Among the current botanical bioherbicide categories of a growing interest include essential oils (EO) that contain several hydrophobic and volatile components (VOCs) and allelochemicals, including a variety of natural herbicide compounds (Macías et al., 2019). Their activity relies on plant-synthesized phytotoxic compounds, which inhibit the root and seedling growth of weeds along with chlorosis, necrosis, or leaf burning (Chang et al., 2022). These arises from various mechanisms effects such as decreased cellular respiration, oxidative damage, and ROS generation, which cause damage to membrane integrity, ion leakage, DNA synthesis and mitosis inhibition, waxy cuticular layer removal, photo-synthesis inhibition, microtubule polymerization, proline accumulation, and lipid peroxidation (Ahuja et al., 2015**;** Lins et al., 2019**;** Chang et al., 2022). Such mechanisms can explain the herbicidal activities of the main allelochemicals and EO, which are encountered in various plants. The action mechanisms of essential oils (EO) and their components (EOc), which have recently gained a growing interest (Acheuk et al., 2022**;** Martini et al., 2023), can illustrate a wide range of botanical bioherbicide activities. For instance, eugenol from clove EO elicits the generation of ROS in plants, leading to cell membrane damage and photosynthesis inhibition (Tworkoski, 2002**;** Bainard et al., 2006**;** Campiglia et al., 2007**;** Ahuja et al., 2015**;** Lins et al., 2019**;** Prasanna et al., 2019). Cinnamic aldehyde from Cinnamomum interacts with the integrated surface receptors that change the ligand-based metabolic pathways (Di Pasqua et al., 2006**;** Lins et al., 2019). Monoterpenes (Cymbopogon), such as citronellal, citronellol, and geraniol can alter the membrane permeability through the phospholipid synthesis regulation, which leads to electrolyte losses (Silva et al., 2016). Pelargonic acid (Pelargonium) has a strong phytotoxic effect superior to that of citronellol, causing the plasma membrane leakage, which, in turn, results in loss of vacuole. Certain β-triketones from plant sources (e.g., *Leptospermum* spp.) inhibit 4-hydroxyphenylpyruvate dioxygenase (HPPD), a key enzyme in the biosynthesis of plastoquinone precursors. Since plastoquinone serves as an essential cofactor for phytoene desaturase, HPPD inhibition results in impaired carotenoid biosynthesis and consequent photooxidative bleaching. This mode of action parallels that of synthetic HPPD-inhibiting herbicides, including mesotrione, tembotrione, and isoxaflutole, which also induce bleaching through carotenoid depletion (Owens et al., 2013).

#### 2.3.3 Microbial and botanical bioherbicide combination

Throughout the literature survey, no information is virtually available on synbiotic bioherbicides that combine live microbes and botanicals, even though great interest in these new weed management options in crops has already been announced (Duke et al., 2022). Three main microbial-botanical combinations can lead to significant herbicidal activities: (1) both components are known as bioherbicides, for instance, by associating compatible bacterial (e.g., *Bacillus* spp.) or fungal (e.g., *Colletotrichum* spp.) and botanical herbicides (e.g., EOc); (2) only one component develops herbicidal activity, e.g. microbial herbicides (e.g., *Bacillus* spp.) formulated with plant-based prebiotics, or bacterial probiotics (e.g., lactic acid bacteria) combined with botanical bioherbicides (e.g., EOc), and (3) none of them exhibits herbicidal activity at all, but their combination becomes active against weeds through synergistic effect. For this third case, the microbial components may transform the botanical one, which becomes active after a bioconversion process. Such cases were observed with antioxidant synbiotics for which Lactobacilli probiotic strains can convert the associated prebiotics into more active compounds (Mounir et al., 2022).

## 3 Synbioherbicide design and preparation strategy

### 3.1 Selection and combination of active components

The first step in synbioherbicide development involves selecting microbial and botanical components based on criteria such as biological activity, safety, and technological suitability. Each potential component is subjected to screening for herbicidal activity, using the in silico, *in vitro*, and/or *in vivo* approach. In addition, the environmental ecotoxicity, host specificity and compatibility with crops, which are critical in developing bioherbicides, must be addressed because they directly determine both safety and effectiveness in real agricultural systems. Bioactive components are applied for targeting weeds without harming crops, non-target organisms, and ecosystems.

A virtual *screening* or in silico approach involves computational evaluation of bioactive agrochemical structures through ligand-receptor modeling using computer-aided pesticide design tools such as Computer-Aided Drug Design (CADD) and Structure-Based Drug Design (SBDD). The goal is to identify the molecular structures with potential herbicidal activity by analyzing physicochemical properties and performing molecular docking. Most structures used in these analyses are sourced from chemical databases or proprietary libraries of synthetic and natural compounds. Numerous potentially herbicidal molecules have been identified using these methods (Walter, 2002**;** Eberhardt et al., 2021).

The *in vitro* approach, also known as the *mechanism-directed* method, focuses on specific molecular mechanisms of herbicidal action. These may imply, for instance, the inhibition of enzymes or proteins, disruption of cell membranes, or interference with hormonal signaling pathways in target weeds. Conversely, the *in vivo* testing employs the classical *phenotypic* method, which assesses the extent and symptoms of phytotoxicity induced by test substances. It often involves pre-emergence seed germination tests in Petri dishes or post-emergence tests on seedlings in growth rooms or greenhouses (Berestetskiy, 2023). On average, more than 150,000 compounds may need to be screened virtually to identify a single lead molecule. Promising candidates selected via molecular docking are then validated through the *in vitro* and/or *in vivo* phytotoxicity bioassays to confirm herbicidal potential (Fu et al., 2019).

### 3.2 Formulation engineering

Formulation is a critical factor in determining both the efficacy and safety of synbioherbicide. Special attention must be given to the properties of active substances, their modes of action, physicochemical characteristics, and the selection of suitable co-formulants (Todero et al., 2018a).

Beyond the active ingredients, co-formulants or adjuvants are added to improve stability, delivery efficiency, and overall product performance. These may include wetting agents, penetrants, solvents, buffers, antimicrobial agents, adhesives, UV protectants, defoamers, and other additives (e.g., inert fillers, dyes, or odorants). Their roles include enhancing shelf stability and ensuring effective field application (Mesnage, 2021).

Herbicide formulations may be either solid—such as powders, granules, or microcapsules—or liquid, including true or colloidal solutions in water or organic solvents, emulsions, and suspensions (Berestetskiy, 2023).

Continuous innovation in formulation types and compositions aims to improve product performance, ease of application, and environmental safety (Tadros Tharwat, 2018). Emerging tools such as artificial intelligence (AI) and advanced silico modeling now enable the virtual screening of numerous formulation scenarios, helping to predict optimal delivery systems and long-term stability profiles (Rajak et al., 2025).

### 3.3 Product controls

Once optimized, the synbioherbicide formulation, either in dry (solid) or wet (liquid) form, undergoes rigorous quality, performance, and stability testing prior to the production scale-up and pilot trials. For dry formulations (e.g., granules and powders), the common quality control (QC) parameters include, for instance, powder color, water activity or relative humidity (Aw), particle size distribution (granulometry), and thermal stability assessed by thermogravimetric analysis (TGA). Powder color monitoring determines the powder’s tendency to brown over time through the browning index (BI) determined by colorimetry, whereas the water activity (Aw) should remain as low as possible (aw < 0.3) to ensure long-term physical, biochemical, and microbiological stability (Kurtmann et al., 2009). Powder particle size data provides an idea on the product homogeneity and stability, which is a quality indicator in relation to the particle aggregation, while its thermal stability measured by the temperature at which the powder starts to decompose/degrade is a critical product heat-resistance. For wet formulations (e.g., dispersion, suspension), typical quality control (QC) metrics include wet particle size distribution, polydispersity index (PDI), and electrophoretic mobility that is an indicator of particle stability in dispersed liquid system against liquid-solid phase separation (Razafindralambo et al., 2019a). These metrics can also serve as fingerprints to monitor and trace over time the powder or dispersion formulation stability. For example, a thermophysical decomposition profile obtained via TGA is an indicator of the powder thermal stability, regarding the storage temperature (Razafindralambo et al., 2019b).

Herbicidal efficacy is assessed through pre-emergence and post-emergence bioassays using target weed species (Song et al., 2024). The survival rate of microbial components can also be evaluated using plate count methods, with results expressed as colony-forming units (CFU) per gram or milliliter of sample.

Shelf life is evaluated through accelerated aging tests conducted under stress conditions such as elevated temperatures. Long-term stability (e.g., over two years) can be predicted using thermo-bacterial stability models (Khalil, 2023), which track key parameters over time—such as droplet diameter d(H) and PDI for liquids, and Aw and color for solids (Berestetskiy, 2023). Viable probiotic enumeration during storage can also be assessed using plate counting method, or through molecular approaches based on propidium monoazide-quantitative PCR (PMA-qPCR) technique, which suppresses amplification of DNA from dead cells and enables strain-specific quantification of viable bacteria in probiotic products (Guo et al., 2024).

**Figure 2:**
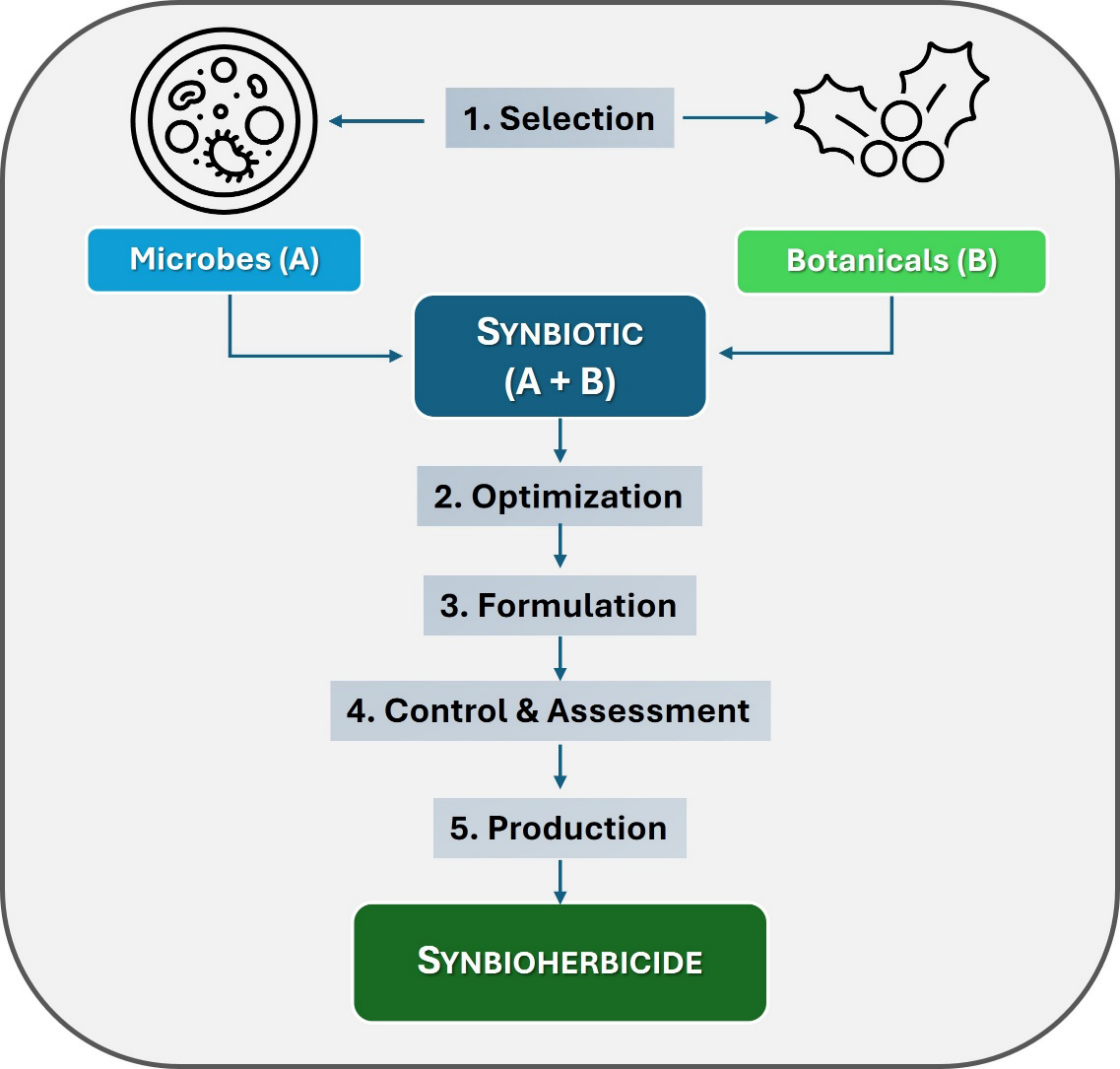
A workflow for the rational design of synbioherbicide.

A workflow for the rational design of synbioherbicide is shown in **Figure 2**. This innovative approach begins with the selection of microbial and botanical components and proceeds through successive steps, including optimization (compatibility, ratios, and doses), formulation, quality control, performance assessment, and finally scale up of production.

The overall goal is to develop a stable and efficacious bioherbicide formulation that exhibits synergistic effects between microbial and botanical components. The optimization step is critical to avoiding antagonistic interactions and to identifying the optimal composition and proportions of each component to maximize synbioherbicide efficacy against the target weed. The selection of appropriate co-formulants and adjuvants, such as (bio)surfactants and (bio)stabilizers, is also essential to product success, ensuring formulation homogeneity, stability, and effective field application.

## 4 Challenges, considerations and potential risks

### 4.1 Efficacy

Most microbial and plant or botanical-based bioherbicide products are recognized as more selective by infecting or interfering with weed growth, whereas synthetic herbicides are more time effective in suppressing target plants (Todero et al., 2018b). Different challenges and barriers are still to breach for replacing or reducing the use of synthetic herbicides by natural ones on a large scale. Among the key factors are high efficiency, large scale production, and competitive price of bioherbicides. Combining microbial and botanical components with synergistic herbicidal activities in synbiotic preparations is therefore a key to promoting new potential bioherbicides in weed management. However, several factors must be overcome to develop synbiotic bioherbicides with high efficacy. Such process involves multiple interactions among active components, but also between active components and targeted weeds, particularly for those of microbial agents – weed cells that are both dynamic living systems. These exhibit intricate physical and biochemical responses, not only to abiotic factors such as temperature, humidity, sunlight, and moisture availability, but also to biotic ones, involving the interactions of all components involved (Hoagland, 1996). This ambitious approach therefore poses multiple challenges and considerations.

First, the selection of ingredients requires relevant, fast, and large screening techniques to cover a wide range of activities, while identifying the bioactive components at the highest microbial taxonomy and chemical structure levels (Duke et al., 2000).

Second, the condition for designing synbiotics is the compatibility of various components involved. The microbial and botanical constituents of synbiotics must be compatible to avoid potential antagonistic effects and ensure at least the maintenance of each component activity. Then, both components are expected to interact synergistically and provide higher herbicidal activity, compared to that of each component used individually.

In fact, many of the major botanicals studied for their herbicidal activity are also recognized as biostatic agents. For example, plant-derived phenolics such as flavonoids, tannins, stilbenes and phenolic acids exhibit herbicidal activity, but can also inhibit the growth and activity of a wide range of microorganisms (Takó et al., 2020). Similarly, terpenes and terpenoids, predominant constituents of essential oils, have been shown to have anti-microbial properties in numerous studies. However, most assessments were performed on the whole EO rather than for each EOc. Thus, identifying relevant and suitable botanicals to incorporate into a synbiotic formulation consists of laborious tasks. Applying the two components separately is an alternative approach for avoiding incompatibility (Hoagland, 1996). For instance, the microbial component(s) can play a helper-driver role (e.g., soil cleaner) before the botanical one with antimicrobial properties at a certain dose accomplishes the herbicidal action. A complementary synbiotic would be at least expected. Another solution to achieve synbiotic application involving incompatible components is to protect one component by using encapsulation technique to avoid antagonistic effects, while controlling the release of the active components (Pimentel-González et al., 2009).

Another aspect to consider is the dose of each component as well as the ratio microbial/botanical. In fact, there exists a potential risk of triggering weed defense mechanisms when using botanicals at sublethal concentrations. This may lead to the elicitation of weed defense responses, which might develop resistance effects towards bioherbicide, depending on the botanical concentration (Hoagland, 1996).

In addition to the discovery of synergistic interactions, the other challenges are the development of stable and effective formulations that maintain the viability and activity of both components, and the application of methods enhancing the efficiency of the synbiotic bioherbicide product. Adjuvants such as (bio)surfactants, emulsifiers and hydrophilic polymers are often used for ensuring efficient formulation by modifying or reinforcing the product physicochemical and functional properties, including particle surface hydrophobicity, granulometry, powder wettability, and dispersibility in dispersing liquid (Ali et al., 2022). The objective is to enhance the spreading, penetration, and absorption of bioactive ingredients in target plants.

Finally, one of the most important challenges in weed management is also to fight weed resistance, which varies from species to others, and is still difficult to control by synthetic herbicides (Holt et al., 2013). Bioherbicides designed in a rational way could have the advantage in minimizing weed herbicide resistance by deploying diverse or/and multiple modes of action (Guo et al., 2020).

### 4.2 Potential risk and barriers

Bioherbicides are considered to have lower toxicity and environmental impact compared to synthetic pesticides, mainly due to their short-lived environmental persistence. However, it is important to recognize that their natural origin does not guarantee complete harmlessness. Some natural toxins that could be used for their phytotoxic activity may also pose a threat to animals, including mammals. It is therefore imperative to thoroughly assess the range of effects of these natural toxins (Takó et al., 2020). For instance, essentials oils are increasingly being studied for their herbicidal properties. However, depending on the dose and composition, some of their compounds can be toxic, allergenic, and mutagenic (Maes et al., 2021). When essential oil compounds exhibit antimicrobial activities, their combination with microbial partners in synbiotic becomes compromised, because of possible incompatibility between these two components. One of suggested solutions in overcoming this inconvenience was to encapsulate one component to avoid the inhibiting activity of the other prior to mixing them. Encapsulation can improve microbial vitality and stability, allowing survival during storage and application. For instance, encapsulation techniques offer several advantages for formulating oil essential-based bioherbicides, as described with many examples (Campos et al., 2023). One of the few studies using encapsulated microbial bioherbicides was the microencapsulation of a fermented broth of *Diaporthe schini* in lactose tested as postemergence treatments against various weeds (Brun et al., 2022). For all species studied, microencapsulated fungi showed better herbicidal activity against *A. viridis* (74%), followed by *B. pilosa* (69%), *Lolium multiflorum* (24%), and *E. crusgalli* (20%) than nonencapsulated fungi.

In addition, some bacterial metabolites such as AAL-toxin produced by *Alternaria alternata* strains were studied for their herbicidal activity but were judged to be too toxic for human and animal health to be used as herbicides (Meena and Samal, 2019).

Another risk in the large-scale production of synbiotic bioherbicides is the feasibility and cost of the processes, especially the biomass production by fermentation and drying techniques. One of the keys is to optimize each process step (Mupondwa et al., 2015). Moreover, scaling-up production while maintaining its herbicidal activity from laboratory to chamber bioassays, or in fields, is not always guaranteed.

### 4.3 Regulatory

From a regulatory perspective, the development and commercialization of bioherbicides is subject to stringent regulatory frameworks. In Europe, the use of microorganisms, chemical mediators, and natural substances is covered by the European Pesticide Regulation (EC) No. 1107/2009 that came into force in 2011 (Cordeau et al., 2016; Robin and Marchand, 2019).

Obtaining the commercial authorization for biocontrol substances is a complex and costly process that has historically posed challenges to novel products (Robin and Marchand, 2019). Notably, new rules were published on 31 August 2022 (Regulation (EU) 2022/1438) that will facilitate the authorization of micro-organisms as active substances in plant protection products (Hoagland, 1996; Harding and Raizada, 2015; Takó et al., 2020). As the concept of synbiotic bioherbicides is new, no regulation is available. However, it is important to notice that when the microbial component is only the active ingredient within the synbiotic preparation, one approval process is necessary, whereas two agreements are needed for synbiotics in which both microbial and botanical components possess herbicidal activities.

Although bioherbicides are generally regarded as more environmentally friendly than synthetic herbicides, they may still pose ecological risks due to the production of toxins or metabolites, environmental persistence, or the infection potential of associated microorganisms. Therefore, the potential environmental hazards of new plant protection products, such as synbioherbicide, must be carefully evaluated through comprehensive ecotoxicological assessments. These evaluations should examine their effects on non-target organisms as well as on key environmental compartments, including soil, water, and air. The primary objective is to ensure that no unacceptable acute or chronic impacts occur within ecosystems following application.

Risk assessment should encompass parameters such as survival, dispersal, infectivity, and pathogenicity of the introduced organisms. In this context, the ecotoxicological risks associated with synbioherbicide composed of beneficial microbes and botanical compounds are expected to be lower when they involve microorganisms and substances classified as generally recognized as safe (GRAS) or included under the qualified presumption of safety (QPS) framework. This is exemplified by organisms commonly used as probiotics, such as lactic acid bacteria (LAB) and certain *Bacillus* strains, as well as by prebiotic compounds including exopolysaccharides and polyphenols.

## 5 Conclusion

In this perspective paper, we propose an innovative concept combining live microbes and botanical components into synbiotic herbicide preparations by considering their potential opportunities, while discussing the most important challenges for their use in weed management. Each synbiotic component may mutually act in complementary or synergism, generating multiple modes of action and diversity, and possible efficacy against resistant weeds. Moreover, such a nature-based “synbioherbicide” category appears as a suitable next generation candidate in contributing to sustainable solutions for agricultural and food security. Despite these opportunities, their development and use require the achievement of stable and effective formulation, which involves, for instance, effective adjuvants and smart encapsulation techniques, but also with a guarantee of large-scale production feasibility and socio-economic viability. Future research should prioritize enhancing the efficacy and scalable production of synbioherbicide while ensuring their safety in controlling weeds, particularly resistant populations. Achieving these goals will require screening a broad range of bioherbicidal components, optimizing their combinations by exploiting synergistic interactions between active and inactive constituents, and refining formulation and delivery systems through the careful selection of co-formulants and adjuvants tailored to specific targets. High-throughput screening, coupled with structure–activity relationship studies using the in silico approaches for compound identification, interaction analysis, and formulation optimization—subsequently validated through experimental work—represents a promising strategy. Such an approach can reduce resource inputs and development timelines, thereby accelerating the commercialization of next generation synbiotic bioherbicides.

## Data Availability

The original contributions presented in the study are included in the article/supplementary material. Further inquiries can be directed to the corresponding author.

## References

[B1] Acheuk F. Basiouni S. Shehata A. A. Dick K. Hajri H. Lasram S. . (2022). Status and prospects of botanical biopesticides in Europe and Mediterranean countries. Biomolecules 12, 311. doi: 10.3390/biom12020311. PMID: 35204810 PMC8869379

[B2] Adetunji C. O. Oloke J. K. Bello O. M. Pradeep M. Jolly R. S. (2019). Isolation, structural elucidation and bioherbicidal activity of an eco-friendly bioactive 2-(hydroxymethyl) phenol, from Pseudomonas aeruginosa (C1501) and its ecotoxicological evaluation on soil. Environ. Technol. Innovation 13, 304–317. doi: 10.1016/j.eti.2018.12.006. PMID: 41869561

[B3] Ahn B. Paulitz T. Jabaji-Hare S. Watson A. (2005). Enhancement of Colletotrichum coccodes virulence by inhibitors of plant defense mechanisms. Biocontrol Sci. Technol. 15, 299–308. doi: 10.1080/09583150400016977. PMID: 41858497

[B4] Ahuja N. Singh H. P. Batish D. R. Kohli R. K. (2015). Eugenol-inhibited root growth in Avena fatua involves ROS-mediated oxidative damage. Pestic. Biochem. Physiol. 118, 64–70. doi: 10.1016/j.pestbp.2014.11.012. PMID: 25752432

[B5] Akhter M. J. Sønderskov M. Loddo D. Ulber L. Hull R. Kudsk P. (2023). Opportunities and challenges for harvest weed seed control in European cropping systems. Eur. J. Agron. 142, 126639. doi: 10.1016/j.eja.2022.126639. PMID: 41869561

[B6] Ali M. A. De Coninck J. Razafindralambo H. L . (2023). “ Wettability of probiotic powders: Fundamentals, methodologies, and applications,” in Wetting and Wettability-Fundamental and Applied Aspects ( IntechOpen, UK).

[B7] Bailey K. L. (2014). “ Chapter 13 - The bioherbicide approach to weed control using plant pathogens,” in Integrated Pest Management. Ed. Abrol D. P. ( Academic Press, San Diego), 245–266. doi: 10.1016/B978-0-12-398529-3.00014-2, PMID:

[B8] Bainard L. D. Isman M. B. Upadhyaya M. K. (2006). Phytotoxicity of clove oil and its primary constituent eugenol and the role of leaf epicuticular wax in the susceptibility to these essential oils. Weed Sci. 54, 833–837. doi: 10.1614/WS-06-039R.1

[B9] Ben Kaab S. Lins L. Hanafi M. Bettaieb Rebey I. Deleu M. Fauconnier M.-L. . (2020). Cynara cardunculus crude extract as a powerful natural herbicide and insight into the mode of action of its bioactive molecules. Biomolecules 10, 209. doi: 10.3390/biom10020209. PMID: 32023949 PMC7072411

[B10] Berestetskiy A. (2023). Modern approaches for the development of new herbicides based on natural compounds. Plants 12, 234. doi: 10.3390/plants12020234, PMID: 36678947 PMC9864389

[B11] Bordin E. R. Frumi Camargo A. Stefanski F. S. Scapini T. Bonatto C. Zanivan J. . (2021). Current production of bioherbicides: mechanisms of action and technical and scientific challenges to improve food and environmental security. Biocatal. Biotransform. 39, 346–359. doi: 10.1080/10242422.2020.1833864. PMID: 41858497

[B12] Boyette C. D. Abbas H. K. (1994). Host range alteration of the bioherbicidal fungus Alternaria crassa with fruit pectin and plant filtrates. Weed Sci. 42, 487–491. Available online at: https://www.jstor.org/stable/4045529 (Accessed October 1, 2025).

[B13] Brun T. Rabuske J. E. Luft L. Confortin T. C. Todero I. Aita B. C. . (2022). Powder containing biomolecules from Diaporthe schini for weed control. Environ. Technol. 43, 2135–2144. doi: 10.1080/09593330.2020.1867651. PMID: 33346723

[B14] Cai H. Gan X. Jin Z. Hao G. (2023). Carboxylic acid derivatives in herbicide development. J. Agric. Food. Chem. 71, 9973–9993. doi: 10.1021/acs.jafc.3c00951. PMID: 37338196

[B15] Campiglia E. Mancinelli R. Cavalieri A. Caporali F. (2007). Use of essential oils of cinnamon, lavender and peppermint for weed control. Ital. J. Agron. 2, 171–178. doi: 10.4081/ija.2007.171. PMID: 41149044

[B16] Campos E. V. R. Ratko J. Bidyarani N. Takeshita V. Fraceto L. F. (2023). Nature-based herbicides and micro-/nanotechnology fostering sustainable agriculture. ACS Sustain. Chem. Eng. 11, 9900–9917. doi: 10.1021/acssuschemeng.3c02282. PMID: 41837259

[B17] Chang Y. Harmon P. F. Treadwell D. D. Carrillo D. Sarkhosh A. Brecht J. K. (2022). Biocontrol potential of essential oils in organic horticulture systems: From farm to fork. Front. Nutr. 8. doi: 10.3389/fnut.2021.805138, PMID: 35096947 PMC8792766

[B18] Cordeau S. Triolet M. Wayman S. Steinberg C. Guillemin J.-P. (2016). Bioherbicides: Dead in the water? A review of the existing products for integrated weed management. Crop Prot. 87, 44–49. doi: 10.1016/j.cropro.2016.04.016. PMID: 41869561

[B19] Dayan F. E. Owens D. K. Watson S. B. Asolkar R. N. Boddy L. G. (2015). Sarmentine, a natural herbicide from Piper species with multiple herbicide mechanisms of action. Front. Plant Sci. 6. doi: 10.3389/fpls.2015.00222, PMID: 25904929 PMC4389368

[B20] De Jong M. D. (2000). The BioChon story: deployment of Chondrostereum purpureum to suppress stump sprouting in hardwoods. Mycologist 14, 58–62. doi: 10.1016/S0269-915X(00)80005-1

[B21] Di Pasqua R. Hoskins N. Betts G. Mauriello G. (2006). Changes in membrane fatty acids composition of microbial cells induced by addiction of thymol, carvacrol, limonene, cinnamaldehyde, and eugenol in the growing media. J. Agric. Food. Chem. 54, 2745–2749. doi: 10.1021/jf052722l. PMID: 16569070

[B22] Duke Dayan Romagni Rimando (2000). Natural products as sources of herbicides: current status and future trends. Weed Res. 40, 99–111. doi: 10.1046/j.1365-3180.2000.00161.x. PMID: 41717205

[B23] Duke S. O. Pan Z. Bajsa-Hirschel J. Boyette C. D. (2022). The potential future roles of natural compounds and microbial bioherbicides in weed management in crops. Adv. Weed Sci. 40, e020210054. doi: 10.51694/AdvWeedSci/2022;40:seventy-five003. PMID: 41739306

[B24] Eberhardt J. Santos-Martins D. Tillack A. F. Forli S. (2021). AutoDock Vina 1.2.0: New docking methods, expanded force field, and python bindings. J. Chem. Inf. Model. 61, 3891–3898. doi: 10.1021/acs.jcim.1c00203. PMID: 34278794 PMC10683950

[B25] Eigharlou M. Hashemi Z. Mohammadi A. Khelghatibana F. Nami Y. Sadeghi A. (2024). Herbicidal proteins from Bacillus wiedmannii isolate ZT selectively inhibit ryegrass (Lolium temulentum L.). Pest Manag Sci. 80, 3478–3490. doi: 10.1002/ps.8053. PMID: 38426586

[B26] Fang W. Liu F. Wu Z. Zhang Z. Wang K. (2022). Plant-associated bacteria as sources for the development of bioherbicides. Plants 11, 3404. doi: 10.3390/plants11233404. PMID: 36501441 PMC9737584

[B27] Frank J. Shakeel A. H. Carballo S. M. Zomorodi S. Pourtaheri P. (2023). Bioherbicides for controlling one or more plant species. Available online at: https://patents.google.com/patent/US20230413828A1/en (Accessed August 7, 2024).

[B28] Fu Y. Zhang D. Zhang S.-Q. Liu Y.-X. Guo Y.-Y. Wang M.-X. . (2019). Discovery of N-Aroyl diketone/triketone derivatives as novel 4-hydroxyphenylpyruvate dioxygenase inhibiting-based herbicides. J. Agric. Food. Chem. 67, 11839–11847. doi: 10.1021/acs.jafc.9b01412. PMID: 31589436

[B29] Gealy D. R. Gurusiddaiah S. Jr A. G. O. (1996). Isolation and characterization of metabolites from Pseudomonas syringae-strain 3366 and their phytotoxicity against certain weed and crop species. Weed Sci. 44, 383–392. doi: 10.1017/S0043174500094042. PMID: 41822556

[B30] Ghorbani R. Leifert C. Seel W. (2005). Biological control of weeds with antagonistic plant pathogens. Adv. Agron. 86, 191–225. doi: 10.1016/S0065-2113(05)86004-3

[B31] Golijan P. J. Sečanski M. Gordanić S. Šarčević T. L. (2023). Weed biological control with fungi-based bioherbicides. Acta agriculturae Serbica 28, 23–37. doi: 10.5937/aaser2355023g

[B32] Gonzini L. C. Hart S. E. Wax L. M. (1999). Herbicide combinations for weed management in glyphosate-resistant soybean (Glycine max). Weed Technol. 13, 354–360. doi: 10.1017/S0890037X00041853, PMID: 41292463

[B33] Graupner P. R. Carr A. Clancy E. Gilbert J. Bailey K. L. Derby J.-A. . (2003). The Macrocidins: Novel cyclic tetramic acids with herbicidal activity produced by Phoma macrostoma. J. Nat. Prod. 66, 1558–1561. doi: 10.1021/np030193e. PMID: 14695796

[B34] Guo Q. Cheng L. Zhu H. Wei L. I. Wei Y. Chen H. . (2020). Herbicidal activity of Aureobasidium pullulans PA-2 on weeds and optimization of its solid-state fermentation conditions. J. Integr. Agric. 19, 173–182. doi: 10.1016/S2095-3119(19)62738-3

[B35] Guo L. Ze X. Jiao Y. Song C. Zhao X. Song Z. . (2024). Development and validation of a PMA-qPCR method for accurate quantification of viable Lacticaseibacillus paracasei in probiotics. Front. Microbiol. 15. doi: 10.3389/fmicb.2024.1456274. PMID: 39171269 PMC11335531

[B36] Harding D. P. Raizada M. N. (2015). Controlling weeds with fungi, bacteria and viruses: a review. Front. Plant Sci. 6. doi: 10.3389/fpls.2015.00659. PMID: 26379687 PMC4551831

[B37] Heap I. (2026). The International Herbicide-Resistant Weed Database. Available online at: www.weedscience.org (Accessed April, 2023).

[B38] Hoagland R. E. (1996). Chemical interactions with bioherbicides to improve efficacy. Weed Technol. 10, 651–674. doi: 10.1017/S0890037X00040586. PMID: 41822556

[B39] Hoagland R. E. Boyette C. D. Stetina K. C. (2023). Bioherbicidal activity of Albifimbria verrucaria (formerly Myrothecium verrucaria) on glyphosate-resistant Conyza canadensis. J. Fungi 9, 773. doi: 10.3390/jof9070773. PMID: 37504761 PMC10381147

[B40] Holt J. S. Welles S. R. Silvera K. Heap I. M. Heredia S. M. Martinez-Berdeja A. . (2013). Taxonomic and life history bias in herbicide resistant weeds: implications for deployment of resistant crops. PloS One 8, e71916. doi: 10.1371/journal.pone.0071916, PMID: 24039727 PMC3767681

[B41] Jiang W. Quan L. Wei G. Chang C. Geng T. (2023). A conceptual evaluation of a weed control method with post-damage application of herbicides: A composite intelligent intra-row weeding robot. Soil Tillage Res. 234, 105837. doi: 10.1016/j.still.2023.105837. PMID: 41869561

[B42] Kennedy A. C. (2018). Selective soil bacteria to manage downy brome, jointed goatgrass, and medusahead and do no harm to other biota. Biol. Control 123, 18–27. doi: 10.1016/j.biocontrol.2018.05.002. PMID: 41869561

[B43] Khalil R. A. (2023). Direct mathematical models for estimating the shelf life of second- and zero-order degradation relationships of food and drugs. Iranian J. Math. Chem. 14, 161–169.

[B44] King R. R. Lawrence C. H. Gray J. A. (2001). Herbicidal properties of the thaxtomin group of phytotoxins. J. Agric. Food. Chem. 49, 2298–2301. doi: 10.1021/jf0012998. PMID: 11368592

[B45] Kolida S. Gibson G. R. (2011). Synbiotics in health and disease. Annu. Rev. Food Sci. Technol. 2, 373–393. doi: 10.1146/annurev-food-022510-133739. PMID: 22129388

[B46] Korres N. E. Burgos N. R. Travlos I. Vurro M. Gitsopoulos T. K. Varanasi V. K. . (2019). “ Chapter six - New directions for integrated weed management: Modern technologies, tools and knowledge discovery,” in Advances in Agronomy. Ed. Sparks D. L. ( Academic Press), 243–319. doi: 10.1016/bs.agron.2019.01.006, PMID:

[B47] Kostina-Bednarz M. Płonka J. Barchanska H. (2023). Allelopathy as a source of bioherbicides: challenges and prospects for sustainable agriculture. Rev. Environ. Sci. Bio/Technol. 22, 471–504. doi: 10.1007/s11157-023-09656-1. PMID: 41868966

[B48] Kouhounde S. Adéoti K. Mounir M. Giusti A. Refinetti P. Otu A. . (2022). Applications of probiotic-based multi-components to human, animal and ecosystem health: Concepts, methodologies, and action mechanisms. Microorganisms 10, 1700. doi: 10.3390/microorganisms10091700. PMID: 36144301 PMC9502345

[B49] Kremer R. J. (2005). “ The role of bioherbicides in weed management,” in Biopestic. Int, vol. 1. ( (Jalandhar) Koul Research Foundation), 4. Available online at: https://www.ars.usda.gov/ARSUserFiles/50701000/cswq-0294-193032.pdf (Accessed September 26, 2007).

[B50] Kubiak A. Wolna-Maruwka A. Niewiadomska A. Pilarska A. A. (2022). The problem of weed infestation of agricultural plantations vs. the assumptions of the European biodiversity strategy. Agronomy 12, 1808. doi: 10.3390/agronomy12081808. PMID: 41725453

[B51] Kurtmann L. Carlsen C. U. Risbo J. Skibsted L. H. (2009). Storage stability of freeze–dried Lactobacillus acidophilus (La-5) in relation to water activity and presence of oxygen and ascorbate. Cryobiology 58, 175–180. doi: 10.1016/j.cryobiol.2008.12.001. PMID: 19111715

[B52] Lamberth C. Jeanmart S. Luksch T. Plant A. (2013). Current challenges and trends in the discovery of agrochemicals. Science 341, 742–746. doi: 10.1126/science.1237227. PMID: 23950530

[B53] Lins L. Dal Maso S. Foncoux B. Kamili A. Laurin Y. Genva M. . (2019). Insights into the relationships between herbicide activities, molecular structure and membrane interaction of cinnamon and citronella essential oils components. Int. J. Mol. Sci. 20, 4007. doi: 10.3390/ijms20164007. PMID: 31426453 PMC6720526

[B54] Macías F. A. Mejías F. J. Molinillo J. M. (2019). Recent advances in allelopathy for weed control: from knowledge to applications. Pest Management Science 75, 2413–2436. doi: 10.1002/ps.5355, PMID: 30684299

[B55] Maes C. Meersmans J. Lins L. Bouquillon S. Fauconnier M.-L. (2021). Essential oil-based bioherbicides: Human health risks analysis. Int. J. Mol. Sci. 22, 9396. doi: 10.3390/ijms22179396. PMID: 34502302 PMC8431140

[B56] Martini F. Jijakli M. H. Gontier E. Muchembled J. Fauconnier M.-L. (2023). Harnessing plant’s arsenal: Essential oils as promising tools for sustainable management of potato late blight disease caused by Phytophthora infestans—a comprehensive review. Molecules 28, 7302. doi: 10.3390/molecules28217302, PMID: 37959721 PMC10650712

[B57] Meena M. Samal S. (2019). Alternaria host-specific (HSTs) toxins: An overview of chemical characterization, target sites, regulation and their toxic effects. Toxicol. Rep. 6, 745–758. doi: 10.1016/j.toxrep.2019.06.021. PMID: 31406682 PMC6684332

[B58] Mesnage R. (2021). “ Coformulants in commercial herbicides,” in Herbicides ( Elsevier), 87–111. doi: 10.1016/B978-0-12-823674-1.00010-9, PMID:

[B59] Mounir M. Ibijbijen A. Farih K. Rabetafika H. N. Razafindralambo H. L. (2022). Synbiotics and their antioxidant properties, mechanisms, and benefits on human and animal health: A narrative review. Biomolecules 12, 1443. doi: 10.3390/biom12101443. PMID: 36291652 PMC9599591

[B60] Muñoz M. Torres-Pagán N. Peiró R. Guijarro R. Sánchez-Moreiras A. M. Verdeguer M. (2020). Phytotoxic effects of three natural compounds: Pelargonic acid, carvacrol, and cinnamic aldehyde, against problematic weeds in Mediterranean crops. Agronomy 10, 791. doi: 10.3390/agronomy10060791. PMID: 41725453

[B61] Mupondwa E. Li X. Boyetchko S. Hynes R. Geissler J. (2015). Technoeconomic analysis of large scale production of pre-emergent Pseudomonas fluorescens microbial bioherbicide in Canada. Bioresour. Technol. 175, 517–528. doi: 10.1016/j.biortech.2014.10.130, PMID: 25459863

[B62] Ofosu R. Agyemang E. D. Márton A. Pásztor G. Taller J. Kazinczi G. (2023). Herbicide resistance: Managing weeds in a changing world. Agronomy 13, 1595–1611. doi: 10.3390/agronomy13061595. PMID: 41725453

[B63] Owens D. K. Nanayakkara N. P. D. Dayan F. E. (2013). In planta mechanism of action of leptospermone: Impact of its physico-chemical properties on uptake, translocation, and metabolism. J. Chem. Ecol. 39, 262–270. doi: 10.1007/s10886-013-0237-8. PMID: 23314892

[B64] Peng G. Wolf T. M. (2011). Synergy between synthetic and microbial herbicides for weed control. Pest Technol. 5, 18–27. Available online at: http://www.globalsciencebooks.info/Online/GSBOnline/images/2011/PT_5(SI1)/PT_5(SI1)18-27o.pdf (Accessed August 19, 2024).

[B65] Pimentel-González D. J. Campos-Montiel R. G. Lobato-Calleros C. Pedroza-Islas R. Vernon-Carter E. J. (2009). Encapsulation of Lactobacillus rhamnosus in double emulsions formulated with sweet whey as emulsifier and survival in simulated gastrointestinal conditions. Food Res. Int. 42, 292–297. doi: 10.1016/j.foodres.2008.12.002, PMID: 41936479

[B66] Prasanna B. Anand A. Anand A. Anand A. (2019). Cinnamon species: *In vivo* anti-oxidant activity of ethanolic extracts of cinnamon zeylanicum and cinnamon cassicae barks. Pharmacognosy J. 11, 245–247. doi: 10.5530/pj.2019.11.38. PMID: 41845114

[B67] Pusztahelyi T. Holb I. J. Pócsi I. (2015). Secondary metabolites in fungus-plant interactions. Front. Plant Sci. 6. doi: 10.3389/fpls.2015.00573. PMID: 26300892 PMC4527079

[B68] Radhakrishnan R. Alqarawi A. A. Abd_Allah E. F. (2018). Bioherbicides: Current knowledge on weed control mechanism. Ecotoxicology Environ. Saf. 158, 131–138. doi: 10.1016/j.ecoenv.2018.04.018. PMID: 29677595

[B69] Rajak B. K. Rani P. Singh N. Singh D. V. (2025). In silico evaluation and simulation-based prioritization of herbicide-like compounds targeting Phalaris minor acetyl-CoA carboxylase. ACS Agric. Sci. Technol. 5, 222–234. doi: 10.1021/acsagscitech.4c00635. PMID: 41837259

[B70] Razafindralambo H. Delvigne F. Blecker C. (2019a). Physico-chemical approach for characterizing probiotics at the solid and dispersed states. Food Res. Int. 116, 897–904. doi: 10.1016/j.foodres.2018.09.026. PMID: 30717021

[B71] Razafindralambo H. Razafindralambo A. Blecker C. (2019b). Thermophysical fingerprinting of probiotic-based products. Sci. Rep. 9, 10011. doi: 10.1038/s41598-019-46469-1. PMID: 31292519 PMC6620332

[B72] Robin D. C. Marchand P. A. (2019). Evolution of the biocontrol active substances in the framework of the European Pesticide Regulation (EC) No. 1107/2009. Pest. Manage. Sci. 75, 950–958. doi: 10.1002/PS.5199. PMID: 30192046

[B73] Shaheen I. Y. Abu-Dieyeh M. H. Ash G. J. Watson A. K. (2010). Physiological characterization of the dandelion bioherbicide, Sclerotinia minor IMI 344141. Biocontrol Sci. Technol. 20, 57–76. doi: 10.1080/09583150903419520. PMID: 41858497

[B74] Shaw D. R. Arnold J. C. (2002). Weed control from herbicide combinations with glyphosate. Weed Technol. 16, 1–6. doi: 10.1614/0890-037X(2002)016[0001:WCFHCW]2.0.CO;2

[B75] Silva C. T. S. Wanderley-Teixeira​ V. Cunha F. M. Oliveira J. V. Dutra K. A. Navarro D. M. A. F. . (2016). Biochemical parameters of Spodoptera frugiperda (J. E. Smith) treated with citronella oil (Cymbopogon winterianus Jowitt ex Bor) and its influence on reproduction. Acta Histochem. 118, 347–352. doi: 10.1016/j.acthis.2016.03.004. PMID: 27012436

[B76] Song K. Ai Y. Zhou J. Dun B. Yue Q. Zhang L. . (2024). Isolation, characterization, and bioherbicidal potential of the 16-residue peptaibols from Emericellopsis sp. XJ1056. J. Agric. Food. Chem. 72, 6315–6326. doi: 10.1021/acs.jafc.3c08984. PMID: 38470442

[B77] Song D. Ibrahim S. Hayek S. (2012). “ Recent application of probiotics in food and agricultural science,” in Probiotics. Ed. Rigobelo E. ( InTech). doi: 10.5772/50121

[B78] Swanson K. S. Gibson G. R. Hutkins R. Reimer R. A. Reid G. Verbeke K. . (2020). The International Scientific Association for Probiotics and Prebiotics (ISAPP) consensus statement on the definition and scope of synbiotics. Nat. Rev. Gastroenterol. Hepatol. 17, 687–701. doi: 10.1038/s41575-020-0344-2, PMID: 32826966 PMC7581511

[B79] Tadros Tharwat F. (2018). Agrochemicals, Paints and Coatings and Food Colloids (Berlin: Walter de Gruyter GmbH&Co KG).

[B80] Takó M. Kerekes E. B. Zambrano C. Kotogán A. Papp T. Krisch J. . (2020). Plant phenolics and phenolic-enriched extracts as antimicrobial agents against food-contaminating microorganisms. Antioxidants 9, 165–165. doi: 10.3390/antiox9020165. PMID: 32085580 PMC7070704

[B81] Tan Z. Han X. Dai C. Lu S. He H. Yao X. . (2024). Functional genomics of Brassica napus: Progress, challenges, and perspectives. J. Integr. Plant Biol. 66, 484–509. doi: 10.1111/jipb.13635. PMID: 38456625

[B82] Todero I. Confortin T. C. Luft L. Brun T. Ugalde G. A. de Almeida T. C. . (2018). Formulation of a bioherbicide with metabolites from Phoma sp. Sci. Hortic. 241, 285–292. doi: 10.1016/j.scienta.2018.07.009, PMID: 41936479

[B83] Tsuge T. Harimoto Y. Akimitsu K. Ohtani K. Kodama M. Akagi Y. . (2013). Host-selective toxins produced by the plant pathogenic fungus Alternaria alternata. FEMS Microbiol. Rev. 37, 44–66. doi: 10.1111/j.1574-6976.2012.00350.x, PMID: 22846083

[B84] Tworkoski T. (2002). Herbicide effects of essential oils. Weed Sci. 50, 425–431. doi: 10.1614/0043-1745(2002)050[0425:HEOEO]2.0.CO;2

[B85] Ulrich A. Müller C. Gasparetto I. G. Bonafin F. Diering N. L. Camargo A. F. . (2023). Bioherbicide effects of Trichoderma koningiopsis associated with commercial formulations of glyphosate in weeds and soybean plants. Crop Prot. 172, 106346. doi: 10.1016/j.cropro.2023.106346. PMID: 41869561

[B86] Uludag A. Uremis I. Arslan M. (2018). “ Chapter 7 - Biological weed control,” in Non-Chemical Weed Control. Eds. Jabran K. Chauhan B. S. (Amsterdam: Academic Press), 115–132. doi: 10.1016/B978-0-12-809881-3.00007-3, PMID:

[B87] Verdeguer M. Sánchez-Moreiras A. M. Araniti F. (2020). Phytotoxic effects and mechanism of action of essential oils and terpenoids. Plants 9, 1571. doi: 10.3390/plants9111571. PMID: 33202993 PMC7697004

[B88] Walter M. W. (2002). Structure-based design of agrochemicals. Nat. Prod. Rep. 19, 278–291. Available online at: https://pubs.rsc.org/en/content/articlehtml/2002/np/b100919m (Accessed October 3, 2025). 12137278 10.1039/b100919m

[B89] Yan Y. Liu Q. Zang X. Yuan S. Bat-Erdene U. Nguyen C. . (2018). Resistance-gene-directed discovery of a natural-product herbicide with a new mode of action. Nature 559, 415–418. doi: 10.1038/s41586-018-0319-4. PMID: 29995859 PMC6097235

[B90] Yin M. Fasoyin O. E. Wang C. Yue Q. Zhang Y. Dun B. . (2020). Herbicidal efficacy of harzianums produced by the biofertilizer fungus, Trichoderma brevicompactum. AMB Express 10, 118. doi: 10.1186/s13568-020-01055-x. PMID: 32613360 PMC7329974

[B91] Zasada I. A. Weiland J. E. Reed R. L. Stevens J. F. (2012). Activity of Meadowfoam (Limnanthes alba) seed meal glucolimnanthin degradation products against soilborne pathogens. J. Agric. Food. Chem. 60, 339–345. doi: 10.1021/jf203913p. PMID: 22142246 PMC4215540

[B92] Zhang Z. Becerra-Alvarez A. Al-Khatib K. (2025). Physiological action of bioherbicides in weed control: A systematic review. Front. Agron. 7. doi: 10.3389/fagro.2025.1633565. PMID: 41869664

